# Electrocardiogram challenge: pericardial effusion

**DOI:** 10.1093/ehjcr/ytaf055

**Published:** 2025-02-07

**Authors:** Sinead Rosser, William Orr, C Fielder Camm

**Affiliations:** Cardiology Department, Royal Berkshire Hospital, London Road, Reading RG1 5AN, UK; Cardiology Department, Royal Berkshire Hospital, London Road, Reading RG1 5AN, UK; Cardiology Department, Royal Berkshire Hospital, London Road, Reading RG1 5AN, UK

A 41-year-old female presented to the emergency department with vomiting and shortness of breath. She has a background history of lung cancer with bone and brain metastases. A CT head showed progressive disease with new cerebral metastases, a chest X-ray showed a large left pleural effusion, and her blood tests showed an acute kidney injury. She was started on i.v. dexamethasone and was awaiting urgent drainage of the pleural effusion. Despite appropriate management, she had ongoing tachycardia of 120 b.p.m., blood pressure 140/100, and a 2L O2 requirement. An electrocardiogram (ECG) was done, which is demonstrated below.

1. What is the ECG finding illustrated in *Figure 1*?
  - ST elevation
  - Electrical alternans
  - Atrial fibrillation
  - Right bundle branch block
  - First degree heart block

**Figure 1:**
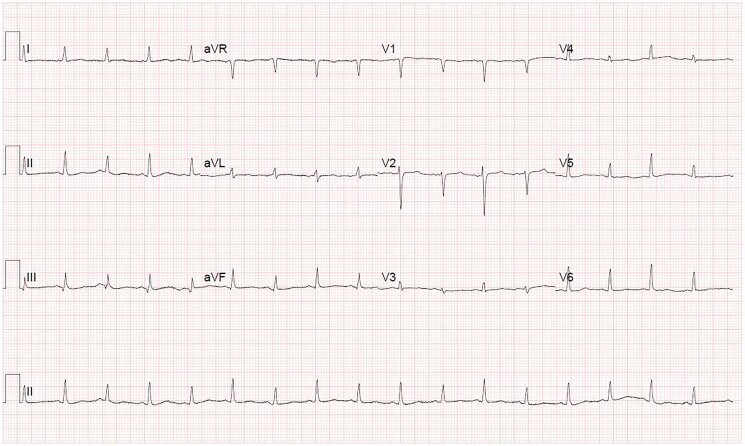
Electrocardiogram.

Correct answer: B) Electrical alternans—electrical alternans is an ECG finding of alternating size of the QRS complex between beats. This can be identified in any or all leads on an ECG.^1^ In the ECG seen above, electrical alternans is most evident in lead V2. Alternating amplitude of the T waves can sometimes be present as well.

2. What pathology results in this ECG finding?
  - Pericardial effusion
  - Pericarditis
  - Myocardial infarction
  - Ventricular septal defect
  - Hypertrophic cardiomyopathy

Correct answer: A) Pericardial effusion—in large pericardial effusions and cardiac tamponade, the heart swings in a pendulum-like motion as it beats, and therefore it alternates between moving towards the ECG electrode, and away from it, hence creating the variation in amplitude of the QRS complexes detected by the ECG and resulting in electrical alternans.^1^ This can be evidenced further in the supplementary video provided (see Supplementary material online, *Video S1*).

3. This patient re-presented one month later with the same pathology. What is the preferred management for long-term prevention of recurrence of this pathology?
  - Repeated pericardial drain insertions
  - i.v. diuretics
  - Chemotherapy
  - NSAIDs
  - Pericardial window

Correct answer: E) Pericardial window—when there is recurrence of pericardial effusion despite pericardiocentesis, the option of a pericardial window should be considered. This can be achieved either surgically or via percutaneous balloon pericardiotomy.^2^ This creates a direct communication between the pericardial sac and the pleural fluid to allow the pericardial fluid to drain and prevent accumulation and tamponade.^3^ Management should also involve addressing the underlying aetiology of the pericardial effusion to prevent recurrence if possible.

## Supplementary Material

ytaf055_Supplementary_Data

## Supplementary material

**Consent:** The patient reported in this case is deceased. Despite the best efforts of the authors, they have been unable to contact the patient's next-of-kin to obtain consent for publication. Every effort has been made to anonymize the case. This situation has been discussed with the editors.

**Funding:** No funding was provided for this work.

**Data availability:** This is a clinical case report where all available data is presented within the manuscript.
